# Estimating animal abundance at multiple scales by spatially explicit capture–recapture

**DOI:** 10.1002/eap.2638

**Published:** 2022-06-29

**Authors:** Eric J. Howe, Derek Potter, Kaela B. Beauclerc, Katelyn E. Jackson, Joseph M. Northrup

**Affiliations:** ^1^ Wildlife Research and Monitoring Section Ontario Ministry of Northern Development, Mines, Natural Resources and Forestry Peterborough Ontario Canada; ^2^ Environmental and Life Sciences Graduate Program Trent University Peterborough Ontario Canada

**Keywords:** American black bear, animal abundance, noninvasive sampling, spatially explicit capture–recapture, survey design, *Ursus americanus*

## Abstract

Information about how animal abundance varies across landscapes is needed to inform management action but is costly and time‐consuming to obtain; surveys of a single population distributed over a large area can take years to complete. Surveys employing small, spatially replicated sampling units improve efficiency, but statistical estimators rely on assumptions that constrain survey design or become less reasonable as larger areas are sampled. Efficient methods that avoid assumptions about similarity of detectability or density among replicates are therefore appealing. Using simulations and data from >3500 black bears sampled on 73 independent study areas in Ontario, Canada, we (1) quantified bias induced by unmodeled spatial heterogeneity in detectability and density; (2) evaluated novel, design‐based estimators of average density across replicate study areas; and (3) evaluated two estimators of the variance of average density across study areas: an analytic estimator that assumed an underlying homogeneous spatial Poisson point process for the distribution of animals' activity centers, and an empirical estimator of variance across study areas. In simulations where detectability varied in space, assuming spatially constant detectability yielded density estimates that were negatively biased by 20% to 30%; estimating local detectability and density from local data and treating study areas as independent, equal replicates when estimating average density across study areas using the design‐based estimator yielded unbiased estimates at local and landscape scales. Similarly, detectability of black bears varied among study areas and estimates of bear density at landscape scales were higher when no information was shared across study areas when estimating detectability. This approach also maximized precision (relative SEs of estimates of average black bear density ranged from 7% to 18%) and computational efficiency. In simulations, the analytic variance estimator was robust to threefold variation in local densities but the empirical estimator performed poorly. Conducting multiple, similar SECR surveys and treating them as independent replicates during analyses allowed us to efficiently estimate density at multiple scales and extents while avoiding biases caused by pooling spatially heterogeneous data. This approach enables researchers to address a wide range of ecological or management‐related questions and is applicable with most types of SECR data.

## INTRODUCTION

Understanding variation in animal abundance is fundamental to applied ecology because this metric is critical for understanding the management and conservation status of species. Ecological processes operate at different scales, which may not coincide with the boundaries of jurisdictions or management units. Thus, methods that can provide accurate estimates at multiple extents provide insight into population dynamics and the effectiveness of interventions (Ferraro & Pattanayak, 2006; Williams et al., 2002). Many species that are the focus of conservation and management programs, including umbrella and flagship species, are also the most difficult and expensive to enumerate because they exist at low and variable densities in habitats that can be difficult to access and travel though, range widely, and avoid human observers (Stokes et al., 2010; Thompson, 2013,). Large areas must be surveyed to obtain representative samples, yet sampling must also be intensive because the animals are often rare, cryptic, or elusive. Months or years of intensive sampling may be required to complete a single survey (Boulanger et al., 2018; Chetri et al., 2019; Howe et al., 2013; Stokes et al., 2010; Tempa et al., 2019); temporally replicated surveys to inform trend can span years or decades (Bisht et al., 2019; Garshelis & Noyce, 2006; Kendall et al., 2019; Walsh et al., 2003).

Wildlife surveys employing spatially replicated sampling units, such as line transects or small arrays (“clusters”) of traps or passive detectors, can improve the efficiency of surveys over large areas by intensively sampling spatial subsets of the area of interest (Buckland et al., 2001; Humm & Clark, 2021; Sollmann et al., 2012; Sun et al., 2014). If sampling units are distributed throughout a predefined region of interest according to a randomized design, design‐based approaches can be used to estimate average density, total abundance, and the variances of these quantities within that region without bias, and without making assumptions about factors that influence density (Buckland et al., 2000; Hankin et al., 2019). Imperfect detection must also be accounted for by statistical models. For example, most distance sampling surveys select locations for point or line transects according to random or systematic designs; density estimators combine model‐based estimation of detectability with design‐based estimation of abundance (Buckland et al., 2001). Sampling locations are assumed to be independent of the density and distribution of animals, so animals cannot be baited or lured towards sampling locations, nor can sampling target habitat features known to be preferred by the species of interest or areas more accessible to researchers to increase sample sizes. Detection is assumed to be certain for objects (animals or their sign) at distance zero from the points or lines (Buckland et al., 2001); provided that this assumption is met, and that areas of varying detectability are sampled proportionally to their occurrence on the landscape, the pooling robustness property ensures that heterogeneity in detectability does not induce bias to estimates of average density across the region of interest (Buckland et al., 2004). Model‐based variances of detectability and empirical variances of encounter rates both contribute to the estimated variance of abundance (Fewster et al., 2009; Thomas et al., 2010). Therefore, distance sampling approaches ensure representative samples, are robust to heterogeneity in detectability, and variance estimators are robust to spatial variation in abundance, but randomized surveys may be inefficient (or logistically infeasible) and animals' reactions to human observers or passive detectors cause bias. These approaches tend to yield imprecise estimates of abundance of rare species, and unreliable estimates for species that react to human observers or passive detectors (Bessone et al., 2020; Buckland et al., 2001; Cappelle et al., 2021; Marini et al., 2009; Plumptre & Cox, 2006).

Currently, many researchers take advantage of our ability to identify individuals from passively collected genetic or photographic samples to enumerate animals that range widely and occur at low densities using spatially explicit capture–recapture (SECR) methods (Arandjelovic & Vigilant 2018; Borchers & Efford, 2008; Boulanger et al., 2018; Després‐Einspenner et al., 2017; Gardner et al., 2009; Greenspan et al., 2020; Humm & Clark, 2021; Linden et al., 2020; Royle et al., 2009, 2013). Studies of American black bears (*Ursus americanus*) in particular have contributed to the ongoing development and testing of SECR methods, and important covariates of detectability were previously identified (Clark, 2019; Gardner et al., 2009, 2010; Humm et al., 2017; Obbard et al., 2010; Sollmann et al., 2012; Sun et al., 2014; Wilton et al., 2014). SECR does not require that locations of individual detectors are independent of the density and distribution of animals, providing greater flexibility and efficiency in survey design and yielding more precise estimates from similar or less survey effort relative to methods that do not require individual identification (Borchers & Efford, 2008; Burgar et al., 2018; Cappelle et al., 2019; Chandler & Royle, 2013; Evans & Rittenhouse, 2018). Estimated densities are specific to the areas in the vicinity of detectors, however, spacing individual detectors widely (e.g., systematically) throughout a large region of interest to improve spatial coverage and representativity is ineffective because designs must ensure that some individuals are detected at more than one location, so the spacing between detectors is constrained by animals' home range sizes (Borchers & Efford, 2008; Sollmann et al., 2012; Sun et al., 2014; Wilton et al., 2014). Clustered designs employing spatially replicated sampling units (small, widely separated arrays of relatively few closely spaced traps or detectors) have been recommended to improve the efficiency of SECR surveys of large areas (Borchers & Efford, 2008; Clark, 2019; Efford et al., 2005; Efford & Fewster, 2013; Sollmann et al., 2012; Sun et al., 2014; Wilton et al., 2014). Designs that yield more detections and especially repeat detections of the same animals (including at different locations) yield more precise estimates (Després‐Einspenner et al., 2017; Sollmann et al., 2012; Sun et al., 2014) and traveling between clusters incurs costs, so researchers may sample more intensively in areas of higher density and areas more accessible to researchers, and target habitat types or features known to be preferred by the species of interest, rather than ensuring that such areas or features are sampled proportionally to their occurrence on the landscape (Fonteyn et al., 2021; Howe et al., 2013; Humm & Clark, 2021; Kolowski & Forrester, 2017; Wilton et al., 2014). If animal densities on sampled areas are not representative of the larger region of interest including the unsampled areas between clusters, estimates of average density and total abundance over that region will be biased (Conn et al., 2017; Wilton et al., 2014). Another potential shortcoming of clustered SECR designs is that individual arrays typically yield insufficient data to estimate model parameters, so data are pooled across replicates for analysis (Azad et al., 2019; Chetri et al., 2019; Howe et al., 2013; Humm et al., 2017; Humm & Clark, 2021; Murphy et al., 2016; Sun et al., 2014; Wilton et al., 2014). However, as larger areas are sampled, it becomes less reasonable to assume that population characteristics such as animal density, detectability, and home range size remain constant in space. This is problematic because heterogeneity in detectability, including spatial heterogeneity, causes negative bias in SECR estimates of abundance unless it is explicitly modeled (Chao, 1987; Gardner et al., 2010; Moqanaki et al., 2021; Sandland & Cormack, 1984; Sollmann et al., 2011; Stevenson et al., 2021). Furthermore, estimators of variance that assume spatially constant density are expected to underestimate if density varies nonrandomly in space (Borchers & Efford, 2008). Therefore, SECR estimates of average density over large areas are prone to bias due to non‐representative sampling and heterogenous detectability, and estimators of variance are prone to underestimation if density varies non‐randomly within the region of interest.

In many analytical frameworks, including both distance sampling and SECR, model‐based approaches can be used to estimate spatially variable abundance as a function of spatial covariates. These approaches do not strictly require random designs or assume spatially constant density, can improve precision, and facilitate the prediction of animal density in areas not sampled (Borchers & Efford, 2008; Miller et al., 2013; Valliant et al., 2000). However, in some situations, it may not be feasible or advisable to estimate spatially variable density as a function of spatial covariates (Conn et al., 2017). Local densities during sampling reflect a recent history of stochastic, potentially interacting bottom‐up and top‐down factors. If these factors are poorly understood, or if spatial covariate data are lacking, error prone, outdated, or collected at an inappropriate scale, hypothesized models of spatial variation in bear density may have little explanatory or predictive power. Formal tests of the goodness of fit of SECR models are not currently available, so analysts cannot confirm that models of spatially variable density provide a good fit to their data even if they are supported over simpler models by model selection criteria. Where appropriate spatial covariates are identifiable and available, both the distribution of covariate values and the relationships between covariates and density should be similar on sampled and unsampled areas or model‐based predictions about densities in unsampled areas will be biased (Valliant et al., 2000).

Over much of the northeastern portion of their range, American black bears are only active within stable home ranges for a matter of weeks before and during the breeding season in spring and early summer, after which they make long distance migrations to patchily distributed, seasonally available food crops, returning to their breeding ranges to den (Noyce & Garshelis, 2011; Obbard et al., 2017). In Ontario, Canada, black bears are sampled during the breeding season to meet the SECR assumption that bears occupy home ranges during sampling (Howe et al., 2013; Obbard et al., 2010), but most weight gain (which is critical to successful reproduction) and most harvest mortality occur in late summer and autumn when bears are traveling widely; harvest data are summarized within management units and bears sampled in one unit are likely to be harvested in a different unit (e.g., Obbard et al., 2017). Therefore, spatial covariates describing habitat productivity and harvest pressure are expected to be poor predictors of local bear densities during sampling. Furthermore, the intensity of human influence on bear habitat is expected to have a large effect on bear density but safety concerns preclude sampling bears at baited detectors near people, so we obtain unbalanced samples with less information from areas where the values of covariates related to human influence are high. Similar constraints may affect surveys of various species, so the potential to combine SECR data and models with design‐based approaches for estimating average abundance over large areas is clearly advantageous.

Research and monitoring programs aimed at species that range over large areas require robust methods for scaling abundance estimates across spatially replicated surveys, thus informing these implications is a critical need in applied ecology. Our main objective was to estimate black bear density at scales relevant to management, while allowing for spatial variation in both detectability and density that was not explicable by available spatial covariates, from logistically feasible and efficient SECR surveys. To that end, we extended the concept of clustered SECR designs to designs where each array of detectors yields sufficient data to estimate local detectability. We describe design‐based estimators of average density and its variance across independent, spatially replicated SECR surveys and evaluate them using simulations and real data from 73 surveys of black bears in Ontario, Canada. Specifically, we used simulations to investigate how robust variance estimators that assume spatially homogenous density are to nonrandom spatial variation in density and to quantify bias caused by ignoring spatial heterogeneity in detectability among replicate surveys. We used our real data to quantify bias caused by residual spatial heterogeneity in detectability when differences among replicate study areas were modeled as additive effects, and to demonstrate the practical advantages of treating surveys as independent replicates during analyses.

## METHODS

### Background: Spatially explicit capture–recapture models

SECR models take advantage of the fact that animals that spend more time near detectors are more likely to be detected. The simplest SECR model requires only two parameters: (1) either the probability of detection (*g*_0_), or the encounter rate (λ_0_), for an animal with a detector placed at its center of activity and (2) the spatial scale over which detection probability or encounter rate declines with increasing distance between animals' activity centers and detectors (σ). The form of the relationship is often assumed to be half‐normal, but other forms can be evaluated. Parameters can be modeled as functions of covariates, and the models can be fitted using either maximum likelihood or Bayesian approaches (Borchers & Efford, 2008; Royle et al., 2013). In the maximum likelihood framework, SECR models can be fitted using either of two likelihood equations: full or conditional (Borchers & Efford, 2008). The full likelihood includes animal density as an estimable parameter, usually as the intensity of a Poisson or binomial spatial point process representing the locations of animals' centers of activity; this allows animal density to be modeled as a function of spatial covariates, and the variance of density to be estimated using standard maximum likelihood approaches (Borchers & Efford, 2008). If one assumes that the point process describing activity center locations is homogeneous (i.e., that animal density is constant, and animals' activity centers are randomly distributed over the area sampled), the likelihood simplifies by conditioning on the number of detected animals (*n*) and density drops out. Fitting the same detection function model using either equation yields identical estimates of the detection parameters (*g*_0_ or λ_0_ and σ). The effective sampling area across all individuals, detectors, and occasions ($\hat{a}$) is estimable as *a* ($\hat{\theta })$, where ($\hat{\theta })$ is the set of maximum likelihood estimates of the detection parameters, including any covariates, which may be specific to individuals, sampling occasions, or detectors. All information about the sampling process, including the number and arrangement of detectors and a potentially complex model describing the detectability and mobility of animals is encapsulated in $\hat{a}$. The expected number of animals within $\hat{a}$ is *n*, so spatially constant density is estimable using the Horvitz‐Thompson‐like estimator $\hat{D}$ = *n* / $\hat{a}$. More generally, when detection parameters depend on covariates (*z*)

(1) $$\hat{D}=\sum_{i=1}^{n}\;\hat{a}{\left(z_{i}\right)}^{-1}$$

where *n* is the number of individuals detected and $\hat{a}$(*z*_*i*_)^−1^ is the effective sampling area for individual *i*. Average density over the entire area sampled by any design, including designs with widely spaced clusters of detectors, is estimable using Equation (1), and covariates of detection parameters may include spatial covariates. For details see Borchers and Efford (2008: 379–80).

### Spatially replicated surveys

When data from different arrays of detectors are treated as independent and analyzed separately, each $\hat{a}$(*z*_*i*_) is specific to the array on which that individual was detected, such that the effective sampling area of all individuals detected on a single array is a function of both individual detectability and sampling effort on array *j*, so Equation (1) no longer applies. We initially considered two estimators of average density across independent arrays (${\hat{D}}_{\text{region}}$): the total number of animals detected divided by the total estimated effective sampling area (Equation 2), and the mean of array‐specific density estimates (Equation 3), where there are *J* independent arrays and *n*_*j*_ and $\hat{a}$_*j*_ denote the number of animals detected, and the estimated effective sampling area, respectively, on the *j*th array.

(2) $${\hat{D}}_{\text{region}}=\frac{\sum_{j=1}^{J}n_{j}}{\sum_{j=1}^{J}{\hat{a}}_{j}}$$

(3) $${\hat{D}}_{\text{region}}=\frac{1}{J}\;\sum_{j=1}^{J}\frac{n_{j}}{{\hat{a}}_{j}}.$$

When $\hat{a}$_*j*_'s are equivalent across arrays, Equations (2) and (3) yield the same result. Equation (2) effectively weights study‐area‐specific $\hat{D}$ by the associated $\hat{a}$_*j*_, which we hoped could accommodate arrays of variable extents. However, when differences in detection parameters cause additional variation in $\hat{a}$_*j*_'s among arrays, arrays with higher detectability inappropriately receive more weight, so ${\hat{D}}_{\text{region}}$ estimated using Equation (2) is expected to be biased (unless heterogeneity in *g*_0_ or λ_0_ and σ is perfectly compensatory; Efford & Mowat, 2014). We suspect that it will rarely be possible, let alone efficient, to ensure that detection parameters are similar across arrays, so we did not consider this estimator further. Equation (3) gives equal weight to each array‐specific $\hat{D}$ regardless of differences in sampling effort or detectability. It is worthwhile to note here that, provided the spacing between arrays ensures that animals have negligible probabilities of being detected on >1 array, estimates of ${\hat{D}}_{\text{region}}$ calculated using Equation (1) from a model with array‐specific detection parameters (i.e., a model with additive differences in *g*_0_ or λ_0_ and σ among arrays and all interactions between array effects and any other covariates) will be equivalent to estimates calculated using Equation (3) from independent analyses of array‐specific data, because then the total effective sampling area is calculated as the sum across the individual‐specific $\hat{a}$(*z*_*i*_)'s, where individuals detected on array *j* have negligible probabilities of being detected at, and do not contribute to the estimated area sampled by, other arrays.

### Estimating variance

Asymptotic variances of $\hat{a}$, and of $\hat{D}$ estimated using Equation (1), were described by Borchers and Efford (2007). Generally, the variance of $\hat{D}$ is estimable as the sum of two components: the variance of $\hat{a}$, and spatial variation in abundance (Borchers & Efford, 2007).

(4) $$\hat{\mathrm{var}}\left(\hat{D}\right)={\hat{D}}^{2}\times \left(\frac{\mathrm{var}\left(n\right)}{n^{2}}+\frac{\mathrm{var}\left(\hat{a}\right)}{{\hat{a}}^{2}}\right).$$

If we treat arrays as independent, sharing no information across arrays when estimating ${\hat{a}}_{j}$'s, then the variance of the estimated total area sampled ($\hat{A}$) is simply the sum of the variances of the array‐specific ${\hat{a}}_{j}$'s

(5) $$\mathrm{var}\left(\hat{A}\right)=\sum_{j=1}^{J}\mathrm{var}\left({\hat{a}}_{j}\right).$$

Assuming spatially constant (homogeneous Poisson) density and substituting Equation (5) into Equation (4) yields

(6) $$\hat{\mathrm{var}}\left({\hat{D}}_{\text{region}}\right)={{\hat{D}}_{\text{region}}}^{2}\times \left(\frac{n}{n^{2}}+\frac{\sum_{j=1}^{J}\mathrm{var}\left({\hat{a}}_{j}\right)}{{\hat{A}}^{2}}\right).$$

Alternatively, we can avoid the assumption of spatially constant density by estimating the empirical variance across replicate arrays (Efford, 2019a). Treating replicate arrays as independent random samples yields the Horvitz‐Thompson‐like estimator:

(7) $$\mathrm{var}\left(n\right)=\frac{J}{J-1}\;\sum_{j=1}^{J}{\left(n_{j}-\frac{n}{J}\right)}^{2}.$$

Substituting Equations (5) and (7) into Equation (4) yields an empirical estimator of the variance of ${\hat{D}}_{\text{region}}$ (Equation 8)

(8) $$\hat{\mathrm{var}}\left({\hat{D}}_{\text{region}}\right)={{\hat{D}}_{\text{region}}}^{2}\times \left(\frac{\;\frac{J}{J-1}\;\sum_{j=1}^{J}\;{\left(n_{j}-\frac{n}{J}\right)}^{2}}{n^{2}}+\frac{\sum_{j=1}^{J}\mathrm{var}\left({\hat{a}}_{j}\right)}{{\hat{A}}^{2}}\right).$$

### Simulations

To assess the performance of the above estimators, and to quantify bias induced by unmodeled spatial heterogeneity in detectability and density, we envisioned a 200 × 300 km rectangular region of interest occupied by black bears, where, unbeknownst to researchers, density (*D*), detectability, or both were either constant, or varied among southern, central, and northern subregions (Appendix S1: Figure S1). We generated activity center locations as homogeneous spatial Poisson point processes distributed throughout either the entire region (constant *D* scenarios), or within subregions (variable *D* scenarios). We simulated sampling of the female segment of the population over six weekly occasions using a design comprising six regular 5 × 8 arrays of 40 proximity detectors at 2‐km spacing. Replicate arrays were themselves distributed in a systematic grid across the entire region of interest, far enough apart that individuals would not be detected at more than one array (Appendix S1: Figure S1).

We simulated sampling assuming detection probability declined according to a half normal function, and that an individual could be detected only once at a location during each occasion. We simulated four scenarios (Table 1): (1) a base scenario where both animal density and detectability were constant, (2) a scenario where density varied threefold among subregions but detectability remained constant, (3) a scenario where g_0_ and σ varied among subregions but density remained constant, and (4) a scenario where both density and detectability varied among subregions (Table 1). Parameter values were selected to approximate estimates from black bears in Ontario (Howe et al., 2013), to be consistent with prior simulation studies (Clark, 2019; Sollmann et al., 2012; Sun et al., 2014), and to ensure that sampling yielded sufficient data to fit SECR models from all scenarios and subregions. Sampling effort was constant across arrays so $\hat{a}$_*j*_ was constant where detectability was constant but varied where detectability varied.

**TABLE 1:** Parameters of spatial capture–recapture models used in simulations

Simulation scenario	*D*	*g*_0_	σ
Constant *D*_*j*_, constant *g*_0_ and σ	12	0.30	1500
Variable *D*_*j*_, constant *g*_0_ and σ	6, 18, 12	0.30	1500
Constant *D*_*j*_, variable *g*_0_ and σ	12	0.25, 0.30, 0.35	1000, 2000, 3000
Variable *D*_*j*_, variable *g*_0_ and σ	6, 18, 12	0.25, 0.30, 0.35	1000, 2000, 3000

*Note*: True values of local (subregion‐specific) density (per 100 km^2^; *D*) and the intercept (*g*_0_) and scale (m; σ) of the half‐normal detection probability function used to simulate sampling in different scenarios. Where more than one value of a parameter is shown the parameter varied among subregions and values are specific to southern, central, and northern portions of the study area, in that order.

We fit an SECR model for proximity detectors (Borchers & Efford, 2008; Efford et al., 2009) with a half‐normal detection probability function and constant *D*, *g*_0_, and σ to data from each array by maximizing the conditional likelihood, and to data pooled across arrays by maximizing the full likelihood. We estimated array‐specific densities (${\hat{D}}_{\text{local}}$) from analyses of array‐specific and pooled data using Equation (1). Models fitted to pooled data assumed constant density so ${\hat{D}}_{\text{local}}$ did not vary among arrays or subregions. We estimated average density across arrays (${\hat{D}}_{\text{region}}$) using Equation (3), using $\hat{a}$_*j*_'s estimated from analyses of both array‐specific and pooled data. We estimated Poisson variances of all estimates of ${\hat{D}}_{\text{local}}$, and of estimates of ${\hat{D}}_{\text{region}}$ from pooled data using Equation (4) following Borchers and Efford (2007). Where ${\hat{D}}_{\text{region}}$ was estimated from independent analyses of array‐specific data, we estimated Poisson variances using Equation (6) and empirical variances using Equation (8). We conducted 1000 iterations of each scenario. We calculated percent relative bias (PRB) of all density estimates and the mean PRB across iterations of each scenario (MPRB). For each estimate of variance, we calculated the relative standard error (RSE) and log‐normal 95% confidence interval of $\hat{D}$, and we report mean relative standard errors (MRSE) and coverage of 95% confidence intervals across iterations. Simulations were performed in R software (version 3.6.2, R Core Team, 2019), using functions implemented in the package *secr* (version 4.1.0, Efford, 2019b).

### Black bears in Ontario, Canada

**FIGURE 1:**
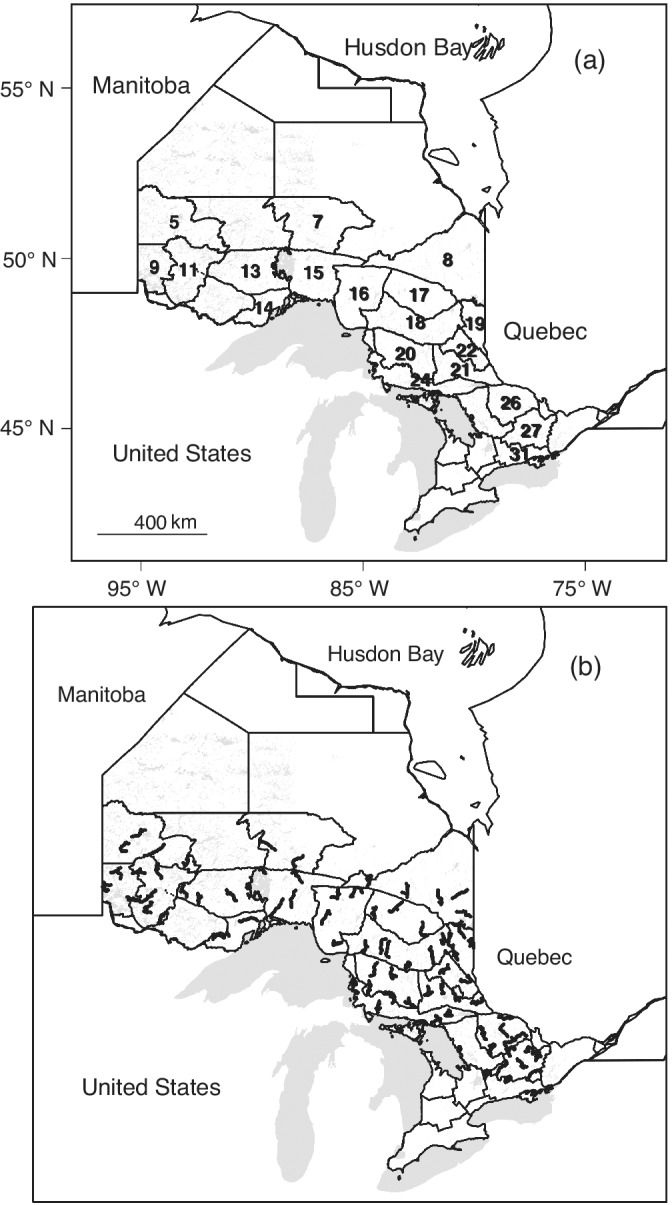
Maps of Ontario, Canada; lakes shown in light gray. Panel (a) shows management zone boundaries; zones where black bears were sampled in 2017, 2018, or 2019 are labeled. Axis labels show longitude and latitude. Panel (b) shows locations of barbed wire hair corrals where black bears were sampled

Black bear range in Ontario was divided into proposed wildlife management zones (MZs; aggregations of the above‐mentioned management units; Figure 1a). Black bears were sampled over five consecutive weekly sampling occasions in spring and early summer of 2017, 2018, or 2019 on 73 curvilinear arrays of approximately 40 baited barbed‐wire hair corrals spaced ~1.5 km apart (some arrays included gaps of up to 6 km to avoid open habitat or human development). Two to six arrays were deployed within each of 12 MZs (Figure 1b). It was not feasible to further subdivide MZs geographically into subregions, or to randomly select locations for the arrays within MZs, due to access limitations (traps were set near [> or ≈ 30 m from] roads or trails to facilitate vehicle [including all‐terrain vehicle] access) and proximity of bear habitat to towns and cities, while keeping entire arrays within one MZ and far enough apart to minimize the chances of detecting individuals at >1 array. We selected curving and branching roads and trails where possible to reduce the potential for bias should home ranges be elongated in a consistent direction with respect to approximately linear arrays (Efford, 2019c). Within these limitations, we attempted to obtain spatially representative samples rather than targeting areas where bears were expected to be most abundant. Arrays in the same MZ were sampled in the same year. The height of the wire strand (~50 cm) was assumed to exclude dependent offspring (cubs and yearlings) from the sample, so detections of different animals were assumed to be independent (conditional on activity center location) and density estimates are considered specific to bears aged >1 year.

Hair samples were air‐dried and stored in paper envelopes prior to DNA extraction. We attempted to genotype all samples consisting of at least five hairs. All extracted samples were genotyped at 15 microsatellite loci and one sex‐specific locus (see Pelletier et al. [2012] for loci, and Appendix S2 for modified laboratory procedures to maximize reliability and efficiency when processing tens of thousands of samples annually). Samples with >14 missing alleles and more than two mixed loci were immediately discarded; remaining samples were grouped into individual genotypes using *allelematch* (Galpern et al., 2012), where the number of allelic mismatches allowed between genotypes was set as 6 to 11. All mismatches were checked; if genotyping error could be confirmed, genotypes were corrected, otherwise the sample was discarded. Individuals represented by single samples required strict criteria for inclusion (Appendix S2). Ambiguities were reamplified and if they could not be verified the sample was discarded. We matched genotypes across samples collected on the same array and subsequently verified that the same 16‐locus genotype was not present on more than one array.

We fit SECR models to array‐specific data by maximizing the conditional likelihood for proximity detectors (Borchers & Efford, 2008; Efford et al., 2009). Regions of integration were defined as the area within a 15‐km buffer around each array and waterbodies were excluded. We fit 15 candidate models of a half‐normal detection probability function to each array‐specific data set (Appendix S1: Table S1). Candidate models included all additive and interactive combinations (including no effects) of location‐specific responses to prior detection (bk) and sex affecting *g*_0_, and additive combinations of sex and temporal variation in σ. We considered two forms of temporal variation: occasion‐specific σ (*t*), and a linear trend in σ on the (log) link scale (*T*). We estimated array‐specific effective sampling areas and densities ($\hat{a}$_*j*_ and ${\hat{D}}_{\text{local}}$, respectively) from AIC_c_‐minimizing models (Akaike information criterion corrected for sample size; Burnham & Anderson, 2002), and their asymptotic variances following Borchers and Efford (2007).

We estimated average bear density across arrays within management zones (${\hat{D}}_{\text{region}}$) using Equation (3), substituting $\hat{a}$_*j*_'s from SECR models that combined information across arrays three different ways. First (1), we treated arrays as independent by using $\hat{a}$_*j*_'s from models fitted to array‐specific data. We also fit multisession SECR models with arrays modeled as sessions to data pooled within MZs and estimated ${\hat{D}}_{\text{region}}$; (2) allowing for additive differences in *g*_0_ and σ among arrays where they were supported by by AIC_c_; and (3) assuming spatially constant detectability. We considered eight candidate models, all of which included a location‐specific response to initial detection affecting *g*_0_ and sex‐specific σ, and which allowed us to evaluate support for differences in *g*_0_ between sexes and among arrays, and differences in σ among arrays (Appendix S1: Table S2). Poisson variances of ${\hat{D}}_{\text{region}}$ were calculated using Equation (6); simulation results (below) indicated that empirical estimates of variance (Equation 8) were unreliable, so we did not estimate empirical variances of ${\hat{D}}_{\text{region}}$ for black bears. Analyses were performed using R version 3.6.1 (R Core Team, 2019), using functions implemented in the *secr* package version 3.2.1 (Efford, 2019a).

## RESULTS

### Simulations

Our base sampling scenario yielded means of 182 animals detected (range 147–231), 552 recaptures (392–775), and 478 spatial recaptures (346–683) from all 240 detectors. In scenarios with varying density and detectability, sample sizes varied with the values of *D*, *g*_0_, and σ simulated. Both density and detectability were low in the southern subregion in scenario 4; array‐specific sample sizes averaged only 10.3 animals (range 1–24), 12.3 recaptures (0–42), and 8.4 spatial recaptures (0–31). In three cases, data were insufficient to fit an SECR model, so we present estimates of local density from analyses of array‐specific data from the 997 iterations of this scenario for which models were successfully fitted to data from all six replicate arrays.

Where neither *D* nor detectability varied among arrays (scenario 1), all estimates of ${\hat{D}}_{\text{local}}$ and ${\hat{D}}_{\text{region}}$ were unbiased (median $\hat{D}$ across iterations = true *D* = 12 per 100 km^2^), and coverage of lognormal 95% confidence intervals calculated assuming spatially homogeneous Poisson density was nominal. Estimates of ${\hat{D}}_{\text{local}}$ from pooled data were more precise (MRSE = 0.08) than estimates from independent analyses of array‐specific data (MRSE = 0.19). In scenarios with spatially variable *D* or detectability, ${\hat{D}}_{\text{local}}$ estimated from independent analyses of array‐specific data was unbiased with nominal confidence interval coverage (Table 2) but estimates of a common ${\hat{D}}_{\text{local}}$ from pooled data were negatively biased relative to the true mean density wherever detectability varied, and was accurate only where detectability was constant and the local density was equal to the regional density (Table 2).

**TABLE 2:** Simulation results: bias, precision, and confidence interval coverage of local density estimates

	Simulation scenarios														
	2: Variable *D*_*j*_, constant *g*_0_ and σ					3: Constant *D*_*j*_, variable *g*_0_ and σ					4: Variable *D*_*j*_, variable *g*_0_ and σ				
	*D*	${\hat{D}}_{\text{local}}$				*D*	${\hat{D}}_{\text{local}}$				*D*	${\hat{D}}_{\text{local}}$			
Median		MPRB	MRSE	CI coverage	Median		MPRB	MRSE	CI coverage	Median		MPRB	MRSE	CI coverage
Independent analyses of array‐specific data^a^															
Grid															
1	6	6	0.33	0.27	0.95	12	12	−0.93	0.24	0.94	6	6	1.29	0.35	0.95
2	6	6	0.90	0.27	0.96	12	12	−1.82	0.24	0.97	6	6	1.66	0.35	0.96
3	18	18	−0.63	0.15	0.96	12	12	−0.70	0.17	0.96	18	18	−0.10	0.14	0.95
4	18	18	−0.66	0.15	0.97	12	12	−0.13	0.17	0.95	18	18	−0.86	0.13	0.96
5	12	12	0.38	0.19	0.94	12	12	−1.00	0.13	0.95	12	12	−0.45	0.13	0.95
6	12	12	−0.92	0.19	0.96	12	12	−0.17	0.13	0.96	12	12	−0.30	0.13	0.94
Analyses of data pooled across arrays^b^															
Grid															
1	6	12	98.75	0.08	0.00	12	9	−27.18	0.067	0.00	6	10	62.49	0.064	0.00
2	6	12	98.75	0.08	0.00	12	9	−27.18	0.067	0.00	6	10	62.49	0.064	0.00
3	18	12	−33.75	0.08	0.00	12	9	−27.18	0.067	0.00	18	10	−45.84	0.064	0.00
4	18	12	−33.75	0.08	0.00	12	9	−27.18	0.067	0.00	18	10	−45.84	0.064	0.00
5	12	12	0.63	0.08	0.95	12	9	−27.18	0.067	0.00	12	10	−18.76	0.064	0.09
6	12	12	0.63	0.08	0.95	12	9	−27.18	0.067	0.00	12	10	−18.76	0.064	0.09

*Note*: True local densities (per 100 km^2^, *D*), median, mean percent relative bias (MPRB), mean relative standard error (MRSE), and confidence interval (CI) coverage of estimated local (array‐specific) densities (${\hat{D}}_{\text{local}}$) across 1000 iterations of each of three simulation scenarios where either density, detectability, or both varied among replicate sampling arrays. The upper panel shows estimates calculated from independent SECR analyses of array‐specific data; the lower panel shows estimates from SECR models fitted to data pooled across arrays, assuming constant detectability and density across arrays.

^a^ No assumptions about spatial variation in *D*, *g*_0_, σ across arrays.

^b^ Fitted model assumed spatially constant *D*, *g*_0_, and σ.

${\hat{D}}_{\text{region}}$ calculated using $\hat{a}$_*j*_'s estimated from pooled data was unbiased in scenarios with spatially constant detectability but severely negatively biased where detectability varied among subregions (Table 3). ${\hat{D}}_{\text{region}}$ calculated using $\hat{a}$_*j*_'s estimated from independent analyses of array‐specific data was unbiased for all scenarios (Table 3), recommending this estimator for use with our real data.

**TABLE 3:** Simulation results: bias, precision, and confidence interval coverage of regional density estimates

			Poisson		Empirical	
Analyses and scenario	Simulation scenario	MPRB	MPRSE	CI coverage	MPRSE	CI coverage
Independent analyses of array‐specific data^a^						
Scenario 1	Constant *D*_*j*_, constant *g*_0_ and σ	−0.6	7.6	0.96	7.3	0.90
Scenario 2	Variable *D*_*j*_, constant *g*_0_ and σ	−0.3	7.7	0.96	19.4	1.00
Scenario 3	Constant *D*_*j*_, variable *g*_0_ and σ	−0.8	6.7	0.93	20.1	1.00
Scenario 4	Variable *D*_*j*_, variable *g*_0_ and σ	−0.4	6.5	0.95	25.1	1.00
Analyses of data pooled across arrays^b^						
Scenario 1	Constant *D*_*j*_, constant *g*_0_ and σ	−0.9	7.6	0.95	7.3	0.90
Scenario 2	Variable *D*_*j*_, constant *g*_0_ and σ	−0.6	7.6	0.95	19.4	1.00
Scenario 3	Constant *D*_*j*_, variable *g*_0_ and σ	**−27**	6.7	0.00	20.1	0.80
Scenario 4	Variable *D*_*j*_, variable *g*_0_ and σ	**−19**	6.4	0.09	25.1	1.00

*Note*: Mean percent relative bias (MPRB; values >1% in bold) of estimated regional densities (${\hat{D}}_{\text{region}}$; female black bears per 100 km^2^) in each of four simulations scenarios, with mean percent relative standard errors (MPRSE) and coverage of 95% lognormal confidence intervals calculated from both analytic (Poisson) and empirical variances. The upper panel shows estimates calculated from independent SECR analyses of array‐specific data; the lower panel shows estimates from SECR models fitted to data pooled across arrays assuming spatially constant density and detectability. Results were derived from 1000 iterations of each scenario, except scenario 4 where results were derived from 997 iterations.

^a^ No assumptions about spatial variation in *D*, *g*_0_, σ across arrays.

^b^ Fitted model assumed spatially constant *D*, *g*_0_, and σ.

MRSE of ${\hat{D}}_{\text{region}}$ calculated from variances estimated assuming an underlying homogeneous Poisson point process for the distribution of activity centers (i.e., assuming constant density across arrays) ranged from 0.06 to 0.08 across simulation scenarios, regardless of whether $\hat{a}$_*j*_'s were estimated from array‐specific or pooled data (Table 3). Coverage of associated 95% confidence intervals was nominal (93%–96% over 1000 iterations) wherever density was estimated without bias, including in scenarios where true *D* varied threefold among subregions (Table 3). The empirical variance estimator underestimated the variance of ${\hat{D}}_{\text{region}}$ in scenario 1 but overestimated uncertainty in scenarios with spatial variation in either detectability or *D* (Table 3), so only the Poisson variance estimator is recommended for use with real data.

### Black bears in Ontario, Canada

Arrays consisted of 40–46 detectors, except for two arrays in fragmented habitat where only 34 and 27 detectors could be deployed. Surveys yielded 54–1691 hair samples, genetic analyses of which revealed 8–111 (mean = median = 49) unique bears detected. Sample sizes of recaptures ranged from 6 to 407 (mean 105, median 87; Appendix S3: Table S1). Seventy of 73 AIC_c_‐minimizing models included a positive location‐specific response to prior detection (bk) affecting *g*_0_ in addition to the effect of sex on σ that was included in all models. Of those 70, 45 also included sex‐specific *g*_0_, and 20 of those included the interaction between bk and sex. Temporal variation in σ was included in 43 AIC_c_‐minimizing models, most often (34 cases) as an increasing linear trend on the link scale. All of these effects received some support across most candidate sets.

Local densities (${\hat{D}}_{\text{local}}$) estimated from array‐specific data ranged from 3 to 36 bears aged >1 year per 100 km^2^ (Appendix S3: Table S2). ${\hat{D}}_{\text{local}}$ varied less than threefold in 13 of 19 MZs, by a factor of approximately three to four in five MZs, and by a factor of six in one MZ (Appendix S3: Table S2). RSEs of ${\hat{D}}_{\text{local}}$ ranged from 13% to 40% of the point estimates (Appendix S3: Table S2). ${\hat{D}}_{\text{local}}$ was not estimable on three arrays with fewer than 10 recaptures (one of six arrays in MZ 27 and both arrays in MZ 31). We estimated ${\hat{D}}_{\text{region}}$ in MZ 27 from independent analyses of the five arrays on which ${\hat{D}}_{\text{local}}$ was estimable, and from data pooled across all six arrays. We did not estimate ${\hat{D}}_{\text{region}}$ from sparse data collected in MZ 31.

In multisession analyses, AIC_c_ consistently supported differences in detection parameters among arrays. Models with differences in *g*_0_, σ, or both *g*_0_ and σ among arrays accounted for 100% of the total AIC_c_ weight across models fit to data from 15 of 19 MZs, and for 94% and 95% of the total weight in two other MZs. Models without such differences were supported only where data were sparse (MZs 27 and 31).

**FIGURE 2:**
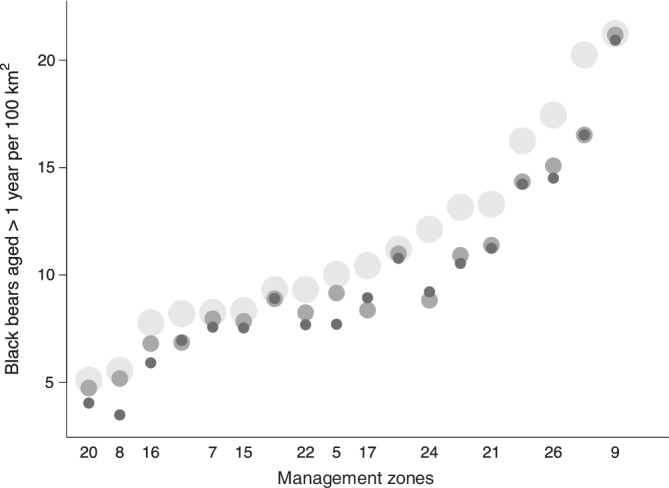
Estimated densities of black bears aged >1 year per 100 km^2^ within each of 18 management zones in Ontario, Canada, 2017–2019. Large, light gray dots show estimates calculated from independent analyses of array‐specific data. Other estimates were calculated from SECR models fit to data pooled across arrays within MZs, allowing for additive differences in detection parameters among arrays (medium‐sized dark gray dots), and assuming spatially constant detectability (small black dots)

**FIGURE 3:**
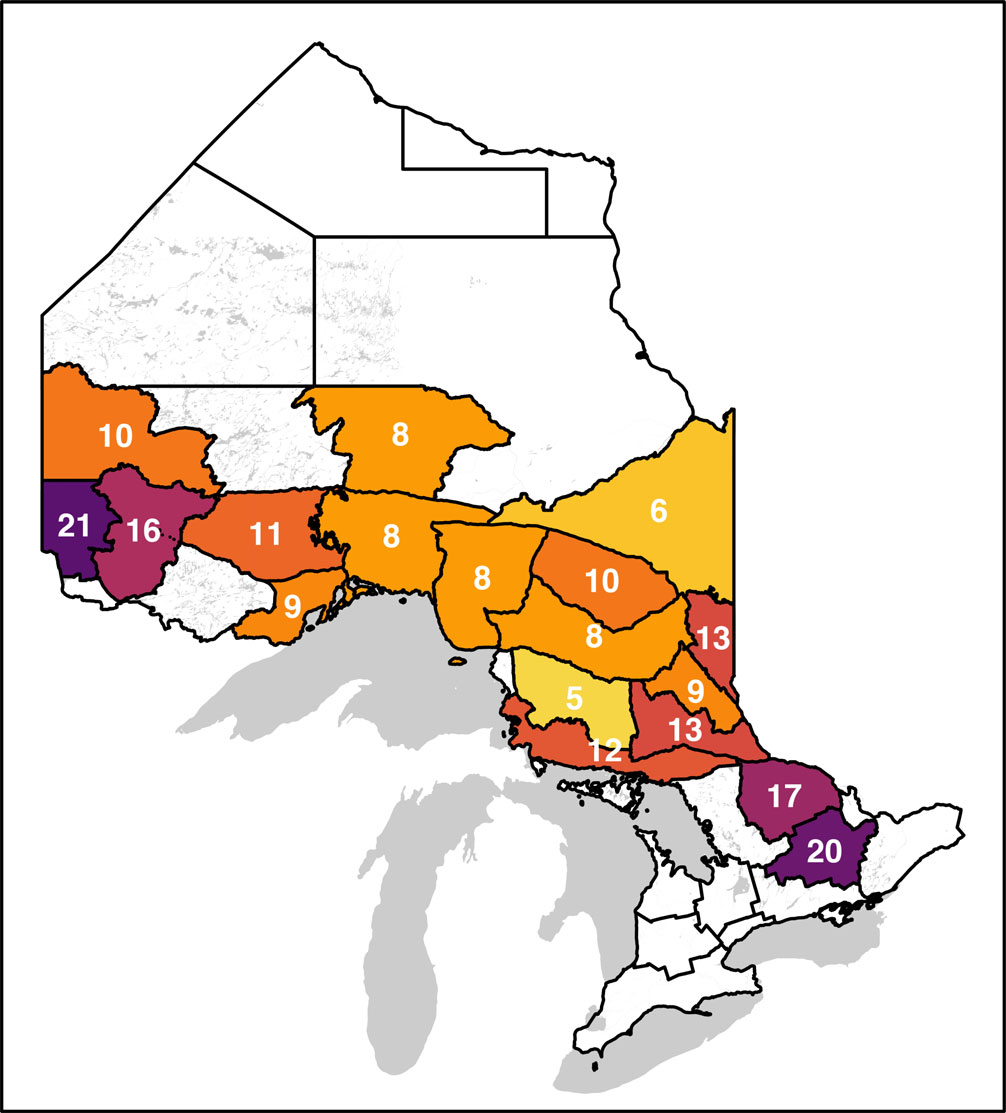
Estimated densities of black bears aged >1 year per 100 km^2^ within management zones calculated from independent analyses of array‐specific data. Colors are specific to the integer values for bear density shown; darker colors indicate higher densities as labeled in white

${\hat{D}}_{\text{region}}$ ranged from 3.5 to 21.2 bears aged >1 year per 100 km^2^; ${\hat{D}}_{\text{region}}$ calculated using Equation (3) from independent analyses of array‐specific data was either similar to or higher than ${\hat{D}}_{\text{Region}}$ calculated using Equation (1) from models fit to data pooled across arrays (Figure 2). ${\hat{D}}_{\text{region}}$ was 1%–37% (mean = median = 16%) lower when we pooled data across arrays and assumed constant detectability than when we treated arrays as independent and equal samples. Modeling differences in black bear detectability among arrays as additive effects reduced but did not eliminate the apparent negative bias: the resulting estimates were still 0%–27% lower (mean 11%, median 12%) than where we treated arrays as independent. Estimates of MZ‐specific average bear densities recommended for management purposes appear in Figure 3; estimates, percent RSEs, and 95% confidence limits are presented in Appendix S3: Table S3).

Where ${\hat{D}}_{\text{region}}$ was estimated from independent analyses of array‐specific data, variances estimated assuming an underlying homogeneous Poisson spatial point process for the distribution of activity centers (Equation 6) yielded RSEs of 10% of the point estimate on average (range 7%–18%; Table 4). Pooling data did not improve precision: pooling and assuming constant detectability yielded RSEs similar to those obtained by treating arrays as independent; pooling and allowing for additive differences in *g*_0_ and σ among arrays yielded the least precise estimates (Table 4).

**TABLE 4:** Precision of regional densities estimated from spatially explicit capture–recapture models fit to array‐specific data, and from two models fit to pooled data

MZ	Independent analyses of array‐specific data	Analyses of data pooled across arrays	
		Model for *g*_0_ and σ across arrays	
		Additive differences	Assumed constant
5	7.4	8.1	8.0
7	9.5	10.3	9.5
8	7.7	10.5	10.0
9	8.6	9.8	8.7
11	7.5	11.8	7.5
13	9.5	11.7	9.4
14	14.8	16.0	15.0
15	8.6	10.2	8.3
16	8.7	12.1	8.7
17	11.0	12.3	11.8
18	7.2	11.2	7.0
19	11.3	14.1	10.7
20	10.8	15.3	11.1
21	10.6	18.4	9.7
22	17.8	15.7	14.4
24	11.7	17.8	9.5
26	10.9	19.7	9.3
27	12.2	11.4	11.4
Mean	10.3	13.1	10.0
Median	10.1	12.0	9.5

*Note*: Percent relative standard errors of estimates of black bear density in each of 18 management zones (MZs) in Ontario, Canada, 2017–2019, derived from variances estimated assuming and underlying homogeneous spatial Poisson point process for the distribution of animals' activity centers within MZs.

## DISCUSSION

Scale (in this case spatial extent) is a fundamental component of wildlife management and conservation decision making. Ecological processes operate across multiple scales (Levin, 1992) and the scale that is most ecologically relevant can vary independent of the social, economic or political scales over which decisions are made (e.g., Robinson et al., 2016), which are also multi‐scale in nature (McDaniels et al., 2006). Thus, the ability to assess the status of wildlife populations across multiple scales simultaneously is critical for monitoring species that range over large and heterogeneous areas. Our results provide a clear and tractable pathway to estimate population density at multiple spatial extents from spatially replicated surveys. We focused on genetic surveys at baited hair corrals, but the approach generalizes to most types of SECR data, including data from live traps, camera traps, or area searches for animals or their DNA. Our methodology also allows for the rapid estimation of average animal density and its variance across any subset of replicate study areas, that is, at multiple scales and over different areas, from SECR models previously fitted to data from different subsets of replicates.

Although we initially assume spatially constant density across replicates to avoid making assumptions about the factors influencing density, sets of spatiotemporally referenced estimates of local density facilitate subsequent investigations of those factors and other relationships of interest. This potential offers substantial opportunity to simultaneously monitor animal populations and describe the mechanisms giving rise to variation in abundance. For example, local density estimates could be treated as the response variable in models with spatial or temporal covariates as predictors. Such “two‐stage” approaches were described in the context of distance sampling surveys: after estimating detectability, plot‐specific counts or abundance estimates are modeled using linear, generalized linear, or generalized additive models (potentially including random effects) to estimate relationships with spatial or temporal covariates and to predict densities throughout the region of interest (Buckland et al., 2015; Hedley & Buckland, 2004). Two‐stage modeling would be much less computationally intensive than simultaneously estimating spatially variable detectability and density using a full‐likelihood or Bayesian SECR model and is a natural extension of our approach of obtaining sufficient data to estimate local detectability and density from replicate‐specific data. Local density estimates can also be used to investigate effects of animal density on other ecological processes, such as predator–prey dynamics.

This is one of the first studies to demonstrate that unmodeled spatial heterogeneity in detectability causes negative bias in SECR estimates of abundance (also see Moqanaki et al., 2021; Stevenson et al., 2021). These results are not surprising because unmodeled heterogeneity in detectability among individuals generally causes negative bias in CR including SECR estimates of animal abundance (Chao, 1987; Link, 2003; Pledger, 2005; Sollmann et al., 2011). Nevertheless, they are an important demonstration for those working at large spatial scales because SECR surveys that use spatially replicated sampling units but devote less effort within replicates (most clustered designs) may yield insufficient data either to estimate cluster‐specific detection parameters or to detect and model differences in detectability among clusters. Our results suggest that such surveys will yield negatively biased estimates if detectability varies among replicate sampling units, and the bias is potentially severe, but will be unknown to the researcher. We were able to avoid this bias only by estimating abundance at all scales from SECR models fitted to replicate‐specific data. Moqanaki et al., (2021) argued that, in addition to heterogeneity that can be accounted for in models or explained using covariates, other local factors may remain unknown. They simulated SECR studies that failed to model spatial heterogeneity in detectability of varying magnitudes and degrees of spatial autocorrelation. Scenarios with high spatial autocorrelation are most similar to our simulations, where detectability was constant across subsets of neighboring detectors. They showed that SECR estimators were robust to many patterns and magnitudes of spatial heterogeneity but yielded negatively biased estimates of abundance when both the magnitude and spatial autocorrelation of variation in detector‐level detectability were high. Ignoring scenarios with “extreme” variation in detectability, negative relative bias approached 30%, similar to our results (table S2.2 of Moqanaki et al., 2021). Together, the results of Moqnaki et al., (2021) and our work suggest that bias is likely present and possibly severe in any studies sampling using a clustered design over any potential variation in density. This should be considered carefully by practitioners when designing studies.

If detectability is similar across groups of animals, pooling data can enhance precision while causing minimal bias (White, 2005); accurate and precise estimates of local density from pooled data in simulation scenario 1, and where local density was equal to the mean density in scenario 2, are examples of such economies of scale. However, in simulations where detectability varied, precision was only enhanced at the expense of bias. Pooling data from black bears did not enhance precision. Regional (MZ‐scale) density estimates were more precise when we analyzed array‐specific data separately than when we allowed for additive differences in detectability among arrays, and of similar precision to when we incorrectly assumed spatially constant detectability across arrays.

In addition to avoiding bias without sacrificing precision, there are practical advantages to the approach we describe for estimating animal abundance over large areas. Local abundances estimated from local data are more meaningful at this scale than estimates derived from a larger data set collected locally and elsewhere (Conn et al., 2006; Howe et al., 2013; White, 2005), and do not change as more replicates are surveyed. Alternatively, if data are pooled and reanalyzed as more replicates are sampled, all local estimates change with the inclusion of the new data, which can cause confusion when communicating results to stakeholders and funding agencies or when rationalizing decision making. Our approach avoids these issues.

Surveys of rare or elusive animals over large areas can take months or years to complete, and trends in abundance of long‐lived species can take years or decades to detect, but funding for wildlife surveys is often variable over short time frames as priorities and budgets shift. If responding to changes in funding or other resources requires a change in sampling methods or survey designs, comparisons in space or time may be compromised. Sampling on multiple independent study areas provides the flexibility to respond to reduced funding simply by sampling on fewer replicates, either by focusing sampling within specific areas of concern, or sampling less intensively across the broader landscape. Replicates could be added as priorities shift back to the species of interest, or to address concerns arising from the introduction of new stressors, while largely maintaining time series of data. For example, black bear sampling scheduled for 2020 was postponed due to COVID‐19, leaving gaps in our coverage of bear range, but this had no effect on our estimates over the areas sampled to date. A final advantage is computational efficiency. Fitting SECR models to large data sets is computationally intensive, requiring access to high performance computing facilities (Howe et al., 2013). Our results suggest that only an SECR model with fully interactive study area effects would eliminate bias caused by pooling spatially heterogeneous data. This approach is mathematically equivalent to fitting models to study‐area specific data, which can be easily accomplished with a stand‐alone computer. Average density across spatial replicates and its variance can subsequently be calculated virtually instantaneously; software to perform the calculations is freely available (Efford, 2019b).

### Limitations

Despite the findings outlined above, there are limitations to both the general approach described here and our application to black bears. We used a design‐based estimator of average density across equal replicates, but locations for real arrays were not randomly selected and we avoided sampling where bears do not occur, so samples are not representative of the total area of management zones. To minimize the associated bias, we attempted to sample representative areas within bear habitat and, when extrapolating to estimate MZ‐specific population sizes for management purposes, we used spatial data describing landcover types to quantify only the area of bear habitat within MZs, excluding non‐habitat. Our recommended estimator of regional density also treated arrays as equal replicates; we attempted to ensure replicates were equal by standardizing array‐specific sampling effort, but areas exposed to sampling actually varied with differences in road networks and therefore array geometry.

Another limitation is the lack of a reliable and theoretically appropriate estimator of the variance of average density across spatially replicated sampling units when density varies in space. The asymptotic (Poisson) estimator is theoretically inappropriate whenever there is extra‐Poisson (non‐random) variation in density. The empirical estimator treats study areas as independent and equal random samples, so is theoretically inappropriate (and performed poorly in simulations) whenever either sampling effort or detectability vary among study areas. In simulations, Poisson variances were surprisingly robust, providing nominal confidence interval coverage even where density differed threefold among subregions. We suggest that the Poisson variance estimator could be used for inference in many situations, acknowledging the potential for underestimation where local density is highly variable among replicates. Unfortunately, our design does not meet the requirements of multi‐stage sampling because the size of our arrays relative to the size of MZs, and logistical constrains on array locations, prevented us from subdividing MZs into subregions and so the sample space and inclusion probabilities remain unknown. In other contexts, multi‐stage designs could allow researchers to apply theoretically appropriate Horvitz‐Thompson‐like estimators of the variance of design‐based estimates of average density across replicate surveys (of Equation 3; e.g., Hankin et al., 2019: 174–181).

To avoid bias due to unmodeled spatial heterogeneity, it is critical to collect sufficient data within each replicate study area to estimate local detectability. From 2004 through 2009, black bears in Ontario were sampled over four or five occasions on multiple arrays of ~25 detectors 2 km apart (Howe et al., 2013; Obbard et al., 2010). Array‐specific data were often sparse, so data were pooled for analysis; results were not finalized until all surveys, genetic analyses, and statistical analyses had been completed; heterogeneity in detectability was apparent in pooled data and remained a concern (Howe et al., 2013; Obbard et al., 2010). Here, surveys with five occasions and ~40 detectors at 1.5 km spacing nearly always provided enough data to estimate local densities with reasonable precision from biologically realistic SECR models fit to exclusively local data.

We acknowledge the potential for unmodeled heterogeneity in detectability in array‐specific data and therefore for negative bias in estimates of black bear density. More detailed, as yet unpublished, analyses of array‐specific data indicate that the finite mixture models most commonly used to accommodate heterogeneity among individuals due to unobserved factors yielded imprecise, potentially unreliable estimates of local density that were highly sensitive to the parameters of the mixture distribution, especially in data sets with fewer detections per individual, which is not surprising given the relatively low probabilities of detecting bears and only five sampling occasions (Dorazio & Royle 2003; Link, 2003; Pledger, 2005).

### Synthesis

Monitoring populations of wide‐ranging, rare, or elusive species at landscape or jurisdictional scales inevitably involves trade‐offs between the cost and feasibility of field surveys, the degree to which surveys satisfy the requirements and assumptions of statistical estimators, and precision. Here, real data from black bears exhibited spatial heterogeneity in detectability and density across replicate study areas; we avoided associated bias in estimates of abundance by collecting sufficient SECR data within each replicate to estimate detectability and abundance locally. Adopting design‐based estimators of average density and its variance across independent replicates allowed us to preserve local estimates of detectability and abundance and maximized precision and computational efficiency when estimating abundance at landscape scales. Survey design constraints prevented us from taking full advantage of the design‐based paradigm. Nevertheless, we suggest that that design‐based or hybrid approaches to estimating abundance by SECR warrant more attention, especially because technological advancements (e.g., in genetic, photographic, and acoustic sampling) will continue to improve our ability to conduct SECR surveys over larger spatial scales.

## CONFLICT OF INTEREST

The authors declare no conflict of interest.

## Supporting information


Appendix S1

Appendix S2

Appendix S3

## Data Availability

Data (Howe, 2021) are available in Dryad at https://doi.org/10.5061/dryad.7wm37pvtz.
