# Supplementary material for: Estimating animal abundance at multiple scales by spatially explicit capture–recapture
Source: Ecol Appl. 2022 Jun 29;32(7):e2638. doi: 10.1002/eap.2638 (PMC9788300; doi:10.1002/eap.2638)
Supplement: Supplementary file 1 — Appendix S1 [file EAP-32-e2638-s002.pdf]

Appendix S1. Supplemental analytical methods

Eric J. Howe, Derek Potter, Kaela B. Beauclerc, Katelyn E. Jackson, Joseph M. Northrup

Estimating animal abundance at multiple scales by spatially explicit capture–recapture

Ecological Applications

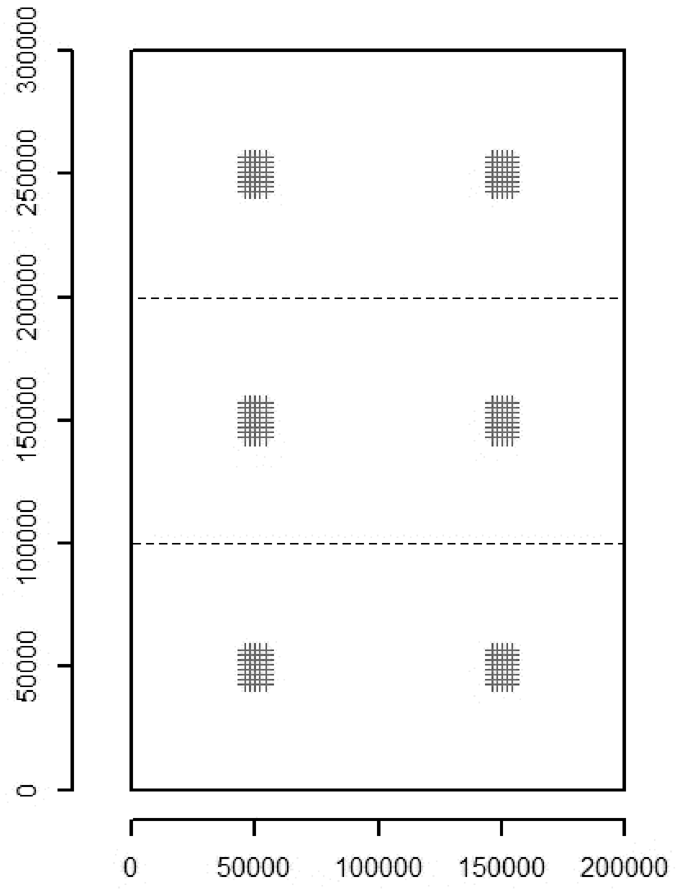

Figure S1. Hypothetical 200 km x 300 km study area from simulations. Southern, central, and northern subregions are separated by dashed lines. Detectors appear as crosses; distances are in m.

Table S1. Candidate spatially explicit capture–recapture models fit to array-specific data from black bears in Ontario, Canada, 2017–2019. Under g_0 and σ, "~*1*" indicates that the parameter was held constant, "*bk*" denotes a trap-specific response to prior detection, "*t*" denotes temporal variation among sampling occasions modelled as a factor, and "*T*" denotes a linear trend across occasions on the (log) link scale. All models assumed a half-normal detection probability function.

Model	g_0	σ
1	~ *1*	~ *sex*
2	~ *bk*	~ *sex*
3	~ *1*	~ *sex* + *t*
4	~ *bk*	~ *sex* + *t*
5	~ *1*	~ *sex* + *T*
6	~ *bk*	~ *sex* + *T*
7	~ *sex*	~ *sex*
8	~ *bk* + *sex*	~ *sex*
9	~ *bk* × *sex*	~ *sex*
10	~ *sex*	~ *sex* + *t*
11	~ *bk* + *sex*	~ *sex* + *t*
12	~ *bk* × *sex*	~ *sex* + *t*
13	~ *sex*	~ *sex* + *T*
14	~ *bk* + *sex*	~ *sex* + *T*
15	~ *bk* × *sex*	~ *sex* + *T*

Table S2. Candidate models of a half-normal spatially explicit capture-recapture detection function fit to data from black bears in Ontario, Canada, (2017–2019), where data were pooled across study areas within management zones.

Model	g_0	σ
1	*bk*	*sex*
2	*bk* + *sex*	*sex*
3	*bk* + *array*	*sex*
4	*bk* +*sex* + *array*	*sex*
5	*bk*	*sex* + *array*
6	*bk* + *sex*	*sex* + *array*
7	*bk* + *array*	*sex* + *array*
8	*bk* +*sex* + *array*	*sex* + *array*
