# Supplementary material for: Estimating animal abundance at multiple scales by spatially explicit capture–recapture
Source: Ecol Appl. 2022 Jun 29;32(7):e2638. doi: 10.1002/eap.2638 (PMC9788300; doi:10.1002/eap.2638)
Supplement: Supplementary file 2 — Appendix S2 [file EAP-32-e2638-s003.pdf]

Appendix S2. Identification of individual black bears from microsatellite genotypes

Eric J. Howe, Derek Potter, Kaela B. Beauclerc, Katelyn E. Jackson, Joseph M. Northrup

Estimating animal abundance at multiple scales by spatially explicit capture–recapture

Ecological Applications

Sample preparation and DNA extraction

Samples were received as hair stored in paper envelopes. Each sample received a unique barcode that was used to track the sample from envelope to genotype. For each sample, all hairs were removed with sterile forceps and placed in an unused, disposable plastic weigh dish. Approximately 20 hairs were separated from the clump, and the remaining hairs returned to the envelope for long term storage at room temperature using silica bead packets to maintain low humidity. For envelopes containing fewer than 20 hairs, all hairs were used. Samples with fewer than 5 hairs were not processed. Otherwise, all samples were processed regardless of hair type, color, quality, etc.

Hairs were wound into a tight ball using two forceps and a novel twirling technique. Each sample was placed at the bottom of a well in a 2mL 96 well plate with 300µL of lysis buffer (2M urea, 0.1M NaCl, 0.25% n-lauroyl sarcosine, 5mM CDTA, 0.05 M Tris HCl pH 8). Each plate contained 4 negative controls (buffers only) and one positive extraction control (300µL subsample from a large volume of previously digested tissue). Once a plate was complete, 20µL of Proteinase K (20mg/mL; Bioshop Canada) was added to each sample, sealed, mixed on a thermomixer at 800rpm for 1min, incubated for 2 hours at 56°C, and stored at 4°C until extraction.

Plates were extracted in batches of four using a modification of the Promega Wizard® SV 96 Genomic Purification System, which uses 96-well plates with silica binding filters and proprietary buffers. Lysate plates were warmed to 56°C for 1hr, 300µL of Wizard® SV Lysis Buffer added to each sample, and mixed at 1300rpm on a thermomixer for 1min at 56°C. All lysate was transferred to a Promega silica binding plate that was placed atop a 1mL deep well plate to collect waste, sealed, and centrifuged at maximum speed (3214g) for 2min. The binding plate was placed on a new waste collection plate, 750µL Column Wash Solution A (Promega) added to each well, and centrifuged at maximum for 2min. This process was repeated twice for a total of 3 washes. A final 5min centrifugation at maximum speed was used to fully dry the silica membrane. The binding plate was then placed on top of a new, labelled, full skirt PCR plate for DNA elution. To each well, 80µL of prewarmed (65°C) $TE_{0.1}$ was dispensed and the plates centrifuged at maximum speed. After elution plates were incubated at 56°C for 2min to evaporate residual ethanol, sealed, and stored at 4°C (if used within 24hr) or -20°C for long term.

Microsatellite genotyping

All extracted samples were genotyped at 15 microsatellite loci and one gender specific locus (amelogenin). Loci are those used by Pelletier et al. (2012) and Obbard et al. (2010), with modifications to maximize reliability and efficiency of genotyping to facilitate processing of >10 000-20 000 samples annually. The 16 loci were amplified with the Qiagen Multiplex PCR Kit in two 12µL multiplex reactions with 2µL stock DNA. Cycling conditions were: 95°C 15min; 30-32 cycles of 94°C for 30s, T_A for 90s, 72°C for 60s; and a final extension of 60°C for 45min. Reaction specific cycling conditions are shown in Table S2.1. One male and one female positive control (250pg each) were amplified in each reaction. PCR product was size separated on an ABI

3730 with Genescan 500 LIZ (Applied Biosystems), and scored using GeneMarker v.2.6.4 (SoftGenetics). All scores were verified by eye and ambiguous alleles were further investigated, reamplified to confirm, or discarded if deemed unreliable as described below.

Table S1. Multiplex conditions for 16 loci using Qiagen Multiplex PCR Kit to genotype black bear samples for individual identification.

Locus	Fluorescent Label	Final concentration (µM)	Reference
Multiplex 1: Mastermix final concentration = 0.8X; T_A = 57°C; 30 cycles			
G10C	FAM	0.2	Paetkeau et al. 1995
G10B	FAM	0.2	Paetkau & Strobeck 1994
G10H	FAM	0.2	Paetkau et al. 1998
G10L	VIC	0.2	Paetkau & Strobeck 1994
G1A	NED	0.22	Paetkau & Strobeck 1994
G1D	PET	0.25	Paetkau & Strobeck 1994
MU05	NED	0.22	Taberlet et al. 1997
AMX/Y	VIC	0.05	Ennis & Gallagher 1994
Multiplex 2: Mastermix final concentration = 1_X; T = 51°C; 32 cycles			
G10X	PET	0.01	Paetkeau et al 1995
G10J	FAM	0.01	Paetkau et al. 1998
G10M	VIC	0.25	Paetkeau et al. 1995
G10P	NED	0.08	Paetkeau et al. 1995
G10U	FAM	0.07	Paetkau et al. 1998
MSUT6	VIC	0.1	Kitahara et al. 2000
MU50	NED	0.1	Taberlet et al. 1997
MU59	NED	0.2	Taberlet et al. 1997

Individual identification of bears

Identification of individuals from genotypes was conducted separately for each trapline. Genotypes were compiled and filtered using a custom R script to remove poor quality samples (more than 14 missing alleles and more than two mixed loci). Remaining samples were grouped into individual genotypes using allelematch in R (Galpern et al. 2012). The number of allelic mismatches allowed between genotypes in a group was set as 6 to 11, according to second zero minimum algorithm as determined in allelematch. The text output was subjected to a custom R script that formatted results to enable rapid visualisation of grouped genotypes and any mismatches between them. All mismatches were checked and corrected if they could be confirmed as genotyping error; if not, the sample was discarded. Homozygous loci were considered reliable if at least 75% of a group of samples had the same genotype. Any individuals represented by a single sample required strict criteria (no mixed loci, at least 10 successfully amplified loci, at least 5 loci with peak heights > 2000RFU) to be considered valid. Any ambiguities were reamplified, and if they could not be verified they were discarded.

All individual bears were given a unique identification. Upon completion of all traplines, all individual bear genotypes were analysed in allelematch. Any bears found across different traplines or years were collapsed into the first unique ID to ensure no false individuals were generated.

References

Ennis, S. & Gallagher, TF. (1994) A PCR-based sex-determination assay in cattle based on the bovine amelogenin locus. Animal Genetics 25(6): 425-7

Galpern, P., Manseau, M., Hettinga, P., Smith, K., & Wilson, P. (2012). Allelematch: An R package for identifying unique multilocus genotypes where genotyping error and missing data may be present. *Molecular Ecology Resources*, *12*(4), 771–778. https://doi.org/10.1111/j.1755-0998.2012.03137.x

Kitahara, E., Isagi, Y., Ishibashi, Y. and Saitoh, T. (2000). Polymorphic microsatellite DNA markers in the Asiatic black bear Ursus thibetanus Microsatellites in the hermaphroditic snail , Lymnaea truncatula , intermediate. *Molecular Ecology*, 1661–1663.

Paetkau, D., Shields, G. F., & Strobeck, C. (1998). Gene flow between insular, coastal and interior populations of brown bears in Alaska. *Molecular Ecology*, *7*(10), 1283–1292. https://doi.org/10.1046/j.1365-294x.1998.00440.x

Paetkau, D., Calvert, W., Stirling, I., & Strobeck, C. (1995). Microsatellite analysis of population structure in Canadian polar bears. *Molecular Ecology*, *4*, 347–354.
