# Supplementary material for: Estimating animal abundance at multiple scales by spatially explicit capture–recapture
Source: Ecol Appl. 2022 Jun 29;32(7):e2638. doi: 10.1002/eap.2638 (PMC9788300; doi:10.1002/eap.2638)
Supplement: Supplementary file 3 — Appendix S3 [file EAP-32-e2638-s001.pdf]

Appendix S3. Supplemental results

Eric J. Howe, Derek Potter, Kaela B. Beauclerc, Katelyn E. Jackson, Joseph M. Northrup

Estimating animal abundance at multiple scales by spatially explicit capture–recapture

Ecological Applications

Table S1. Summaries of data collected during 73 genetic spatially explicit capture–recapture surveys of black bears in Ontario, Canada, 2017–2019, showing the management zone (MZ) sampled, and numbers of hair traps, hair samples collected, individuals identified, total detections (limited to one per individual per location per sampling occasion), total recaptures, and the mean number of detections per animal (DPA) on each array. Means and medians at bottom are across the survey-specific values shown, not across all traps or animals.

Year	MZ	Traps	Samples	Individuals	Detections	Recaptures	DPA
2017	13	40	576	48	131	83	2.7
2017	13	41	1199	71	293	222	4.1
2017	13	40	596	40	148	108	3.7
2017	15	41	1522	67	350	283	5.2
2017	15	40	1566	69	315	246	4.6
2017	15	40	773	53	165	112	3.1
2017	18	40	507	32	145	113	4.5
2017	18	40	1152	61	319	258	5.2
2017	18	40	604	53	160	107	3.0
2017	18	40	639	51	196	145	3.8
2017	18	40	643	39	136	97	3.5
2017	18	40	592	42	142	100	3.4
2017	19	40	128	23	38	15	1.7
2017	19	41	432	45	125	80	2.8
2017	19	40	590	49	137	88	2.8
2017	19	40	458	38	108	70	2.8
2017	21	40	399	34	95	61	2.8
2017	21	40	480	53	118	65	2.2
2017	21	40	562	61	146	85	2.4
2017	21	40	531	57	144	87	2.5

Table S1 continued.

Year	MZ	Traps	Samples	Individuals	Detections	Recaptures	DPA
2018	5	40	1023	82	246	164	3.0
2018	5	40	604	52	132	80	2.5
2018	5	40	1224	74	307	233	4.1
2018	5	40	934	49	251	202	5.1
2018	8	40	740	37	193	156	5.2
2018	8	40	328	33	96	63	2.9
2018	8	40	682	51	173	122	3.4
2018	8	40	847	52	194	142	3.7
2018	8	40	1038	58	251	193	4.3
2018	11	41	921	64	217	153	3.4
2018	11	40	1181	75	253	178	3.4
2018	11	40	791	67	176	109	2.6
2018	11	40	683	51	155	104	3.0
2018	11	40	817	62	139	77	2.2
2018	17	40	530	38	145	107	3.8
2018	17	40	422	61	128	67	2.1
2018	17	40	363	51	101	50	2.0
2018	20	41	270	24	57	33	2.4
2018	20	40	273	27	68	41	2.5
2018	20	40	533	46	133	87	2.9
2018	20	40	681	37	190	153	5.1
2018	22	40	658	44	143	99	3.3
2018	22	40	449	42	94	52	2.2
2018	26	40	807	81	190	109	2.3
2018	26	46	143	25	44	19	1.8
2018	26	40	317	44	72	28	1.6
2018	26	40	579	59	141	82	2.4
2018	26	40	367	40	77	37	1.9
2018	26	42	348	39	72	33	1.8

Table S1 continued.

Year	MZ	Traps	Samples	Individuals	Detections	Recaptures	DPA
2019	7	40	1691	78	485	407	6.2
2019	7	40	1038	64	266	202	4.2
2019	9	40	725	59	191	132	3.2
2019	9	40	717	70	155	85	2.2
2019	9	40	1354	111	298	187	2.7
2019	14	40	496	66	134	68	2.0
2019	14	40	335	33	97	64	2.9
2019	16	40	512	42	121	79	2.9
2019	16	41	820	64	271	207	4.2
2019	16	40	649	52	196	144	3.8
2019	16	41	831	40	189	149	4.7
2019	24	40	331	32	97	65	3.0
2019	24	40	317	33	85	52	2.6
2019	24	40	532	50	144	94	2.9
2019	24	40	262	35	95	60	2.7
2019	24	40	363	45	100	55	2.2
2019	27	40	412	43	100	57	2.3
2019	27	40	291	45	76	31	1.7
2019	27	40	358	50	90	40	1.8
2019	27	40	146	22	29	7	1.3
2019	27	42	264	33	59	26	1.8
2019	27	40	193	28	53	25	1.9
2019	31	34	54	8	14	6	1.8
2019	31	27	212	19	25	6	1.3
Minimum		27	54	8	14	6	1.3
Maximum		46	1691	111	485	407	6.2
Mean		40	622	49	154	105	3.0
Median		40	576	49	141	87	2.8
Total		2918	45405	3573	11219		

Table S2. Densities of black bears aged >1 year per 100 km^2 on each of 70 independent study areas (arrays of detectors) in Ontario, Canada, 2017–2019, estimated from the AIC$_c$-minimizing of 15 candidate spatially explicit capture–recapture models fitted to each study area-specific data set, with standard errors (SE's), percent relative standard errors (PRSE), and upper and lower 95% confidence limits (LCL and UCL).

MZ	Density	SE	PRSE	LCL	UCL
5	14	1.9	14	11	18
5	10	1.7	18	7	14
5	12	1.7	14	9	16
5	5	0.8	17	3	6
7	9	1.5	16	7	13
7	7	1.0	14	5	10
8	5	1.1	24	3	7
8	8	1.4	17	6	12
8	5	0.9	19	3	7
8	4	0.6	16	3	5
8	6	1.0	16	4	8
9	25	3.2	13	19	32
9	13	2.3	17	9	18
9	26	4.5	18	18	36
11	10	1.8	18	7	15
11	14	2.5	18	10	20
11	19	3.8	19	13	28
11	18	2.6	14	14	24
11	19	3.6	19	13	27
13	14	2.6	19	10	20
13	8	1.5	19	5	11
13	12	1.7	14	9	16
14	14	2.7	20	9	20
14	5	1.1	23	3	8
15	9	1.3	14	7	12
15	7	1.1	15	5	10
15	9	1.8	20	6	13
16	7	1.5	21	5	11
16	10	2.0	20	7	15
16	5	0.8	15	4	7
16	9	1.6	19	6	13

Table S2 continued

MZ	Density	SE	PRSE	LCL	UCL
17	5	0.9	19	3	7
17	17	4.0	23	11	27
17	9	1.9	21	6	14
18	18	2.9	16	13	24
18	7	1.2	18	5	10
18	3	0.7	20	2	5
18	7	1.5	21	5	11
18	8	1.1	15	6	10
18	6	1.4	21	4	10
19	16	5.1	33	8	29
19	12	3.0	25	7	20
19	18	3.0	17	13	25
19	7	1.5	21	5	11
20	6	1.4	21	4	10
20	3	0.6	19	2	5
20	5	1.3	28	3	8
20	6	1.5	24	4	10
21	10	2.1	20	7	15
21	14	2.9	20	9	21
21	21	4.4	21	14	32
21	8	1.6	21	5	12
22	8	1.4	18	5	11
22	11	3.9	36	6	21
24	8	2.1	26	5	13
24	14	4.2	29	8	25
24	5	1.3	28	3	8
24	14	2.7	20	9	20
24	19	4.1	21	13	29
26	26	4.7	18	18	37
26	15	4.9	32	8	28
26	13	2.3	18	9	18
26	11	3.0	27	7	19
26	8	3.1	40	4	17
26	31	9.4	30	18	56
27	11	2.6	24	7	17
27	27	8.3	31	15	48
27	36	11.1	31	20	65
27	10	3.6	35	5	20
27	17	3.4	20	12	25

Table S3. Estimated densities, with percent relative standard errors (PRSE) and upper and lower 95% lognormal confidence limits (LCL, UCL), of black bears aged >1 year per 100 km^2 ($\widehat{D}$) in each of 18 management zones (MZs) in Ontario, Canada (2017–2019), estimated using Eq. 3 from independent analyses of array-specific data from 2 to 6 arrays in each MZ. PRSEs and lognormal confidence limits were calculated from variances estimated assuming an underlying homogeneous Poisson spatial point process for the distribution of activity centers.

MZ	arrays	$\widehat{D}$	PRSE	LCL	UCL
5	4	10.0	7.4	8.7	11.6
7	2	8.3	9.5	6.9	9.9
8	5	5.5	7.7	4.8	6.5
9	3	21.2	8.6	17.9	25.1
11	5	16.2	7.5	14.0	18.8
13	3	11.2	9.5	9.3	13.5
14	2	9.3	14.8	7.0	12.4
15	3	8.3	8.6	7.0	9.9
16	4	7.8	8.7	6.6	9.2
17	3	10.4	11.0	8.4	12.9
18	6	8.2	7.2	7.1	9.4
19	4	13.2	11.3	10.6	16.4
20	4	5.1	10.8	4.1	6.3
21	4	13.3	10.6	10.8	16.4
22	2	9.3	17.8	6.6	13.2
24	5	12.1	11.7	9.8	15.1
26	6	17.4	10.9	14.1	21.6
27	5	20.2	12.2	16.0	25.7
